# The Prethalamus Is Established during Gastrulation and Influences Diencephalic Regionalization 

**DOI:** 10.1371/journal.pbio.0050069

**Published:** 2007-03-06

**Authors:** Nicole Staudt, Corinne Houart

**Affiliations:** Medical Research Council Centre for Developmental Neurobiology, King's College London, London, United Kingdom; Cambridge University, United Kingdom

## Abstract

The vertebrate neural plate contains distinct domains of gene expression, prefiguring the future brain areas. In this study, we draw an extended expression map of the rostral neural plate that reveals discrete domains inside the presumptive posterior forebrain. We show, by fate mapping, that these well-defined cell populations will develop into specific diencephalic regions. To address whether these early subterritories are already committed to restricted identities, we began to analyse the consequences of ablation and transplantation of these specific cell populations. We found that precursors of the prethalamus are already specified and irreplaceable at late gastrula stage, because ablation of these cells results in loss of prethalamic markers. Moreover, when transplanted into the ectopic environment of the presumptive hindbrain, these cells still pursue their prethalamic differentiation program. Finally, transplantation of these precursors, in the rostral-most neural epithelium, induces changes in cell identity in the surrounding host forebrain. This cell–non-autonomous property led us to propose that these committed prethalamic precursors may play an instructive role in the regionalization of the developing diencephalon.

## Author Summary

During the earliest stages of development, the brain is first formed as a simple sheet of cells called the neural plate. Although the plate looks homogenous, it contains distinct domains that can be identified by differential gene expression. These domains correspond to distinct future brain areas. In this study, we examined gene expression patterns in an area of the neural plate that later forms the forebrain to show that well-defined cell populations will develop into specific forebrain regions, such as the prethalamus, thalamus, hypothalamus, and epithalamus. We then tested whether these early neural plate subterritories are fully committed to a particular forebrain identity. We found that precursors of the prethalamus are not replaceable by other neighbouring cells, because ablation of these cells results in loss of prethalamus development. Moreover, when prethalamus precursors were moved into the environment of the presumptive hindbrain, the cells still pursued their prethalamic differentiation program. Finally, when the prethalamic precursors were moved to areas of the future forebrain, they transformed the surrounding host forebrain. We propose that the committed prethalamic precursors play an instructive role in the regionalization of the developing forebrain.

## Abstract 3

This study shows that prethalamic identity is established as early as the end of gastrulation, thereby elucidating the developmental stage at which prethalamus identity is assigned to a specific cell population inside the neural plate.

## Introduction

The vertebrate brain is divided caudal to rostral into the hind-, mid-, and forebrain. These territories are further developing into highly specialised structures. In the case of the hindbrain, subdivisions are easy to detect morphologically, because rhombomeres are marked by visible borders during the course of development [1]. By contrast, the partition of the embryonic forebrain is not as obvious. The optic recess creates a border between the dorso-rostral telencephalon and the diencephalon. The latter is traditionally split into five different domains: the ventrally located hypothalamus, the prethalamus (or ventral thalamus), the thalamus (or dorsal thalamus), the dorsally positioned epithalamus, and the caudalmost pretectum [2].

The prosomeric model by Puelles and Rubenstein [3] suggests that the forebrain develops from a set of compartments called prosomeres. Boundaries between individual prosomeres are not morphologically visible. It is hypothesized, based on gene expression profiles, that the pretectum, the thalamus, and the prethalamus develop from different prosomeres [3].

Inside the embryonic diencephalon, morphological landmarks distinguish the forming hypothalamus and epithalamus. The zona limitans intrathalamica (ZLI) separates the prethalamus from the thalamus and pretectum, and has been shown recently to be required for the maintenance of prethalamic and thalamic identities [1,4]. The molecular and cellular events leading to this complex organization are very poorly understood. Until recently, the neural plate itself was considered as a uniform epithelium of unspecified neural precursors. However, the detection of local signalling centres inside the forming neural plate [2] indicates that, as the neural plate forms, at least some cell populations are strictly committed to distinct fates [5,6]. An increasing number of genes found to be expressed in restricted areas inside the rostral neural plate raise the possibility that a whole set of forebrain fate decisions are already taken inside this nascent neuroepithelium.

To gain knowledge of early forebrain development, fate map studies are of great interest. Fate maps of the rostral neural plate have been generated in frog, chick, and mouse [7–10]. These studies show that precursors of a given forebrain region are located in a very defined area of the neural plate, implying very little or no cell mixing during early brain development. In zebrafish and medaka, fate maps were generated at early, mid, and late gastrulation stages [11–14]. These fish neural plate maps establish the broad location of forebrain, midbrain, and hindbrain precursors during neural plate maturation without reaching a resolution revealing the location of different diencephalic areas inside the neural plate.

Being predominantly interested in exploring cell-fate specification inside the presumptive diencephalon, we set out to generate detailed expression and fate maps of the diencephalic anlage in the mature neural plate (bud stage in zebrafish). We constructed our expression map by double in situ hybridization using a vast panel of forebrain markers. In addition, we established a refined fate map of the anterior neural plate using a laser uncaging technique. The results show a remarkable segregation of each major diencephalic domain. Finally, we show, using ablation and transplantation approaches, that one such domain, the presumptive prethalamus, is already strictly committed and irreplaceable inside the neural plate, and exerts some control upon fate decision of its surroundings. All together, our observations strongly suggest the existence of a novel neural plate “mid-forebrain” compartment, regulating early steps of forebrain partitioning.

## Results

### At Neural Plate Stage, the Presumptive Diencephalon Contains Distinct Domains of Gene Expression

To date, studies of gene expression in the vertebrate anterior neural plate have highlighted the presence of, from rostral to caudal, a horseshoe-shaped telencephalic domain, followed by a well-defined eye field, which in turn abuts a diencephalic anlage (see [2] for review). Our previous work suggested that the ablation of at least one restricted population of diencephalon precursors (row 6/7 [6]) could lead to defects in posterior forebrain development, opening the possibility that some irreversible cell-fate decisions may be taken inside the posterior forebrain territory at that time [6]. To elucidate the diencephalic organization at such an early stage of development, we first established a rostral neural plate gene expression map.

**Figure 1:**
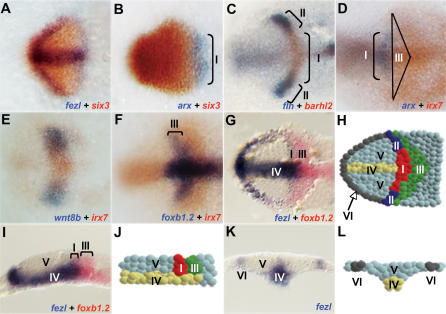
Expression Profile of the Rostral Neural Plate (A–G) Rostral neural plate, anterior to the left. Embryos at bud stage (A–D, F, G , I, and K) or 100% epiboly (E) are shown. (A) *six3* (red), *fezl* (blue). (B) *six3* (red), *arx* (blue). (C) *barhl2* (red), *flh* (blue). (D) *irx7* (red), *arx* (blue). (E) *irx7* (red), *wnt8b* (blue). (F) *irx7* (red), *foxb1.2* (blue). (G) *foxb1.2* (red), *fezl* (blue). (H) Scheme of the different expression domains inside the diencephalon anlage shown in (A–G). (I) Sagittal section, anterior left, *foxb1.2* (red), *fezl* (blue). (J) Scheme of (I). (K) Transverse section, *fezl* (blue). (L) Scheme of (K). The different domains are the presumptive anteromedial (I), anterolateral (II), and posterior (III) diencephalon, hypothalamus (IV), and eye field (V).

We found that, at bud stage, diencephalic markers like *arx* [15], *fezl* [16], and *barhl2* [17] are located directly posterior to the eye field (Figure 1A and 1B). Inside that domain, *flh* [18] is exclusively expressed laterally (domain II in Figure 1C, and unpublished data), overlapping with the telencephalic territory; whereas *arx* is expressed in the medial part of the *barhl2* expression domain at the same developmental stage (domain I in Figure 1B and 1C). In cells posterior to the ones expressing *arx,* transcripts of *irx7* [19] are detected (domain III in Figure 1D). Although *irx7* has been described to be expressed in the midbrain and hindbrain, an overlap of the rostral expression domain with that of the forebrain marker *pax6a* [20] shows that the anterior-most *irx7-*expressing cells are located within the presumptive forebrain territory (unpublished data). Other genes that we found to be expressed in the posterior diencephalic domain III are *wnt8b* (Figure 1E) and *foxb1.2* (Figure 1F and 1G), which are expressed in an overlapping pattern with *irx7* (Figure 1F) and are located posterior to *fezl* (Figure 1G). The resulting expression map of early forebrain markers shows a subdivision of the forebrain into six different domains (Figure 1H), which expands the previously described map of the rostral neural plate, revealing subdomains inside the diencephalic anlage. Besides the hypothalamic anlage (domain IV in Figure 1) the diencephalon is further partitioned into the anterior medial domain I *(arx, fezl,* and *barhl2),* the anterior lateral domain II *(flh* and *barhl2),* and the posterior domain III *(irx7, foxb1.2,* and *wnt8b).*

Sagittal (Figure 1I and 1J) and transverse (Figure 1K and 1L) sections of embryos showing *fezl* (blue) and *foxb1.2* (red) expression indicate that, by bud stage, hypothalamic cells are positioned underneath the eye field. This can be interpreted in two ways. Either the neural plate has already begun to keel and the presence of a seemingly continuous retinal field is due to invisible ventricular limits; or, alternatively, the neural plate is not strictly a single pseudo-stratified epithelium, and the hypothalamic precursors are lying, and moving, underneath a single eye field. This second interpretation is supported by previously published fate maps in axolotl [21], *Xenopus* [10], and chick [22] showing an eye field uninterrupted by hypothalamic precursors; and by a very recent study, showing by computer-aided cell-tracking experiments [23] that, contrary to what has been previously suggested [13], the eye field is not split by hypothalamic precursors in zebrafish.

### Differential Dynamic Shape Changes in Rostral and Caudal Diencephalic Anlagen

**Figure 2:**
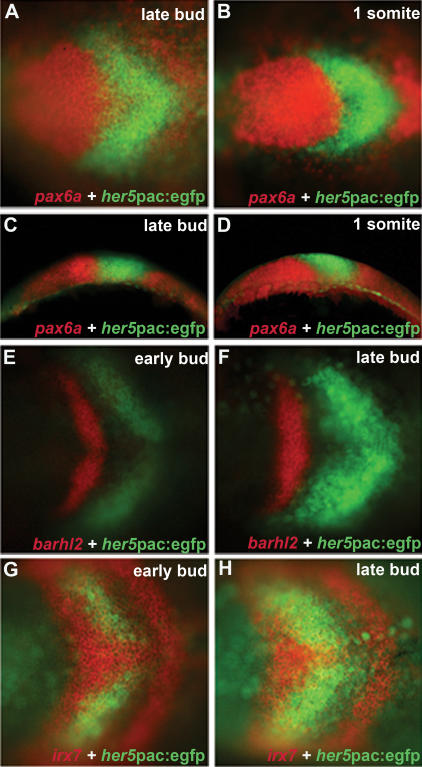
GFP Transgenic Lines Provide Good Landmarks in the Neural Plate (A–D) *pax6a* (red) and *her5*pac:egfp (green) are expressed in adjacent neural plate territories at bud stage (A and C) and 1-somite stage (B and D); The same embryos are shown in dorsal (A and B) and lateral views (C and D), rostral to the left. (C and D) show dynamic changes in the neural plate positioning the rostral midbrain dorsal to the caudal diencephalon; (E and F) Dorsal views, rostral to the left of neural plates showing *barhl2* (red) and *her5*pac:egfp (green) expression revealing a shape change of the rostral diencephalon from a mild V-shape (E) at early bud stage to a straight line (F) at late bud stage; (G and H) Dorsal views, rostral to the left of neural plates showing *irx7* (red) + *her5*pac:egfp (green) expression, visualizing the domain of the caudal diencephalon anterior to the midbrain at early bud stage (G) and late bud stage (H).

To assess whether the different diencephalic subdomains found in our expression map give rise to specific areas of the developing forebrain, we set out to perform fate-mapping experiments. To achieve a high level of precision in labelling neural plate cells, we needed visible landmarks inside the rostral neural plate. We made use of the green fluorescent protein (GFP) transgenic line *her5*pac:egfp [24], which shows a very robust GFP expression in a V-shaped territory covering the anlagen of the midbrain and rostral hindbrain at bud stage. Tallafuss and Bally-Cuif [24] showed that the most rostrally positioned GFP-expressing cells contribute exclusively to the developing midbrain. By combination with the forebrain marker *pax6a,* we confirm that *her5*pac:egfp is expressed directly posterior to the presumptive forebrain inside the neural plate (Figure 2A–2D). Testing the location of the different diencephalic expression domains in combination with the GFP marker, we see a clear gap between the anterior diencephalic markers such as *barhl2* and GFP (Figure 2E and 2F). Inside this gap, the expression of the posterior marker *irx7* is found (Figure 2G and 2H). The mild V-shape expression domain of the anterior diencephalon anlage at early bud stage (Figure 2E) has straightened by late bud stage (Figure 2F). Conversely, the anlage of the midbrain still remains V-shaped, revealing a triangular posterior diencephalic territory at that stage. These shape changes are accompanied by the initiation of keel formation (neurulation), which positions the rostro-alar midbrain area on top of the posterio-basal diencephalon during early somitogenesis (Figure 2D). As a consequence, at later stages of embryogenesis, the rostral part of the dorsal midbrain (optic tectum) appears to lie dorsal to the caudal-most ventral diencephalon (anterior tegmentum).

### Specific Cell Populations inside the Rostral Neural Plate Give Rise to Distinct Regions of the Developing Diencephalon

**Figure 3:**
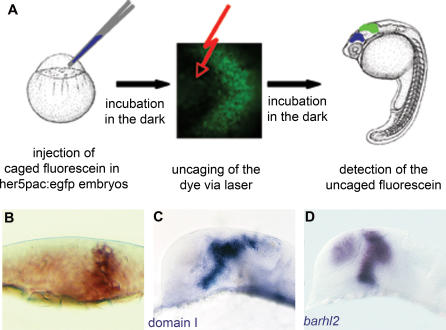
Schematic of the Uncaging Procedure (A) Caged fluorescein is injected into one-cell stage *her5*pac:egfp embryos. Injected embryos are kept in the dark until the fluorescein is uncaged in 6–10 cells of the diencephalic neural plate at bud stage using a laser beam. After further light-protected incubation, the embryos are fixed at prim5 stage, and the uncaged form of the fluorescein is detected via antibody staining. (B) Transverse section of a neural plate after the uncaging experiment, cells of the whole *z*-axis are labelled. (C) Result of labelling cells via uncaging in domain I, which correlates mostly with the expression pattern of *barhl2* at bud stage. (D) At prim5, labelled areas in the diencephalon resemble the endogenous expression pattern of *barhl2*.

Our expression map of the presumptive forebrain shows that cells located in the diencephalic territory differ already by their molecular constituents. To assess whether these molecular subdivisions match territories fated to become well-defined parts of the developing diencephalon, we performed fate-mapping studies of these specific cell populations, using the *her5*pac:egfp transgenic fish (permitting a high level of accuracy in cell targeting). We injected a caged form of fluorescein into one-cell stage *her5*pac:egfp transgenic embryos. Once the embryos reached bud stage, the fluorescein in 6–10 cells, within arbitrary domains, were uncaged using a laser beam (Figure 3A; see Materials and Methods). A cross section of the neural plate just after uncaging shows that cells have been labelled all along the *z*-axis (Figure 3B), indicating that our setup allows precision along the *xy*-axes, but not control of depth inside the neural plate. When uncaging broad areas of the diencephalic neural plate, we observe that cells inducing a specific early diencephalic marker tend to keep expressing it specifically through somitogenesis. Indeed, when we carefully label the *barhl2* expression domain at bud stage, fluorescein is detected in a broad forebrain domain at prim 5, which closely resembles the expression pattern of *barhl2* in the diencephalon at the same stage (Figure 3C and 3D).

**Figure 4:**
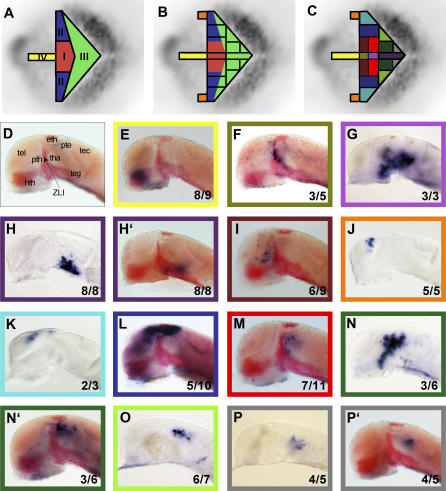
Fate of Caudal Forebrain Precursors (A) Schematic showing the four different diencephalic expression domains identified in Figure 1. (B) Arbitrary subdivision grid of the caudal forebrain shown on top of the expression domains plus an additional telencephalic subdomain (orange). (C) Colour code used for the final 12 domains investigated. Embryos in (D, E, F, H′, I, L, M, N′, and P′) show the expression of *shh* and *otx5* in red. (D) Different areas of the rostral brain are labelled on an embryo showing expression of *shh* (in the ZLI) and *otx5* (in the pineal gland) in red. (E) Cells of the bright yellow midline area are found in the hypothalamus. (F) Cells inside the lime midline domain populate the ventral ZLI. (G) The light violet area spans the posterior hypothalamus, the basal ZLI, posterior tuberculum, and some of the anterior tegmentum. (H and H′) Cells of the caudal-most midline domain of the forebrain are detected in the anterior tegmentum. (I) Cells labelled in the dark red domain of the neural plate contribute to the prethalamus. (J) Precursors of the orange area will populate the posterior dorsal-most telencephalon. (K) The light blue includes precursors of the dorsal telencephalon and the epithalamus. (L) The dark blue area marks cells of the dorsal telencephalon, epithalamus, and dorsal pretectum. (M) In the light red area, we found precursors of the thalamus. (N–P) (N and N′) Cells of the dark green domain can be found in the dorsal thalamus and, in some cases, in a stream of cells running down to the posterior hypothalamus (N′). Precursors of the light green area (O) populate parts of the pretectum and the tectum whereas cells of the grey area (P and P′) were detected in cells close to the midbrain. Because of the nature of the experiment (negative landmarks and fast developing embryos), we were not always reproducibly labelling the same cells in each experiment. Therefore, one can find, for each experiment, the number of embryos we labelled per experiment and how many of these result in the shown cell labelling. (For example 8/9 means eight out of nine embryos show the same labelling in the area depicted in the picture). eth, epithalamus + pineal gland; hth, hypothalamus; pte, pretectum; pth, prethalamus; tec, tectum; teg, tegmentum; tel, telencephalon; tha, thalamus.

Because we wanted to establish a more precise fate map of the diencephalon, we decided to label smaller areas of the neural plate using an arbitrary grid. Figure 4A shows a more abstract version of the expression map shown in Figure 1, highlighting domains I to IV. The correlation of the chosen grid and the expression map is represented in Figure 4B in which we also added an additional (orange) domain to address the border between the diencephalon and the telencephalon. After a set of preliminary data using the grid on Figure 4B, we refined our arbitrary domains to 12 colour-coded areas (Figure 4C). For a better understanding of the results, we indicated different areas of the rostral brain in a prim5 embryo displaying *shh* expression (Figure 4D). The outcome of the uncaging experiments is shown in Figure 4E–4P′; the colour of the frames corresponds to the colour-coded grid in Figure 4C. Forebrain cells of the anterior midline populate reproducibly the developing hypothalamus (Figure 4E) and the eye field (not shown in our dissected brains), whereas cells posterior to those give rise to cells in the posterior tuberculum and anterior tegmental domains (Figure 4G–4H′). At the interphase between these two domains, we found a medial cell population displaying an unexpected behaviour. These cells, located inside the anterior diencephalic anlage revealed by the expression map, give rise to progenies that spread into a more-alar region than their caudal and rostral midline neighbours (Figure 4F). This domain corresponds to the ventral-most part of the presumptive ZLI (co-localises with the *shh*-expressing cells, Figure 4F). Cells of the dark red–labelled region in Figure4C become the prethalamus (Figure 4I). Close to this area, towards the edge of the neural plate (orange and light blue area), precursors of the dorsal telencephalon (Figure 4J) or dorsal telencephalon and epithalamus (Figure 4K) are found. When we labelled cells in a slightly more medial position (dark blue), the fluorescein was detected in a region spanning the dorsal telencephalon and the epithalamus, and abutting the dorsal pretectum (Figure 4L). Cells of the bright red and dark green areas are destined to become part of the thalamus (Figure 4M–4N′). And cells positioned in the neural plate very close to our GFP landmark give rise to the most-caudal forebrain cells (pretectum and anterior tegmentum, Figure 4O–4P′).

**Figure 5:**
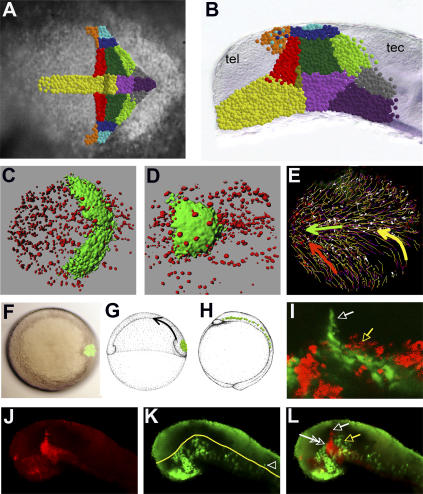
Cell Movement in the Neural Plate (A and B) Schematic overview of the results obtained by the fate-mapping experiments. Colours at prim5 stage correspond to the territories labelled at bud stage. (C–E) Time-lapse analysis of nuclei (red) movement in the neural plate in *her5*pac:egfp (green): 3D rendering of a 200-μm *z*-series done at (C) bud stage and (D) 5-somite stage. (E) Schematic overview of the trajectories of individual nuclei over the recorded time (time-lapse movie can be seen in Video S1), with labelled nuclei in white. Wavy lines and dark to light colours of the lines represent the timeline of nuclei movement. Some lines are shorter because nuclei moved out of or into the observed area over the recorded time. We observed three types of movement: the basal cells move anteriorly (green arrow), posterior alar cells move towards the midline and then anteriorly (yellow arrow), and anterior alar cells move diagonally towards the midline (red arrow). (F–L) Transplantation of basal cells: (F) animal pole view in which labelled cells (green) are transplanted on top of the shield; (G) schematic lateral view of shield stage embryo in which transplanted cells are going to move towards the animal pole during gastrulation; (H) schematic lateral view of bud stage embryo in which transplanted cells are spread along the midline of the embryo; and (I) *shh*GFP is shown in green, transplanted cells in red, at 22 hpf in a live embryo. (J–L) *shh* is shown in red, transplanted cells in green; at 30 hpf, basally derived cells form a large proportion of the brain, showing that just the tip of the ZLI is formed by alar plate cells (white arrow), the basal ZLI is composed of basal cells (white double arrow), and the ventral part of the developing thalamus is built by basal cells (yellow arrow). The yellow line in (K) indicates the border between alar and basal plate; the arrowhead in (K) points to a single basal cell moving alar.

If one compares the relative distribution of the subdomains at bud stage and at prim5 (Figure 5A and 5B), one can predict that a simple rostral shift of the midline accompanying the closure of the plate into a keel may be sufficient to create most of the embryonic brain pattern observed at prim5. To investigate whether such a shift takes place and if so, at which stage of development this movement happens, we used confocal microscopy to create time-lapse movies allowing to monitor the movement of neural plate cells from bud stage to mid-somitogenesis. We recorded *her5*pac:egfp transgenic embryos in which a proportion of the nuclei were labelled with red fluorescent protein (RFP; see Materials and Methods). With this technique, we were able to follow cells of the diencephalic territory during the course of development (Video S1). After three-dimensional (3D) reconstruction (Figure 5C and 5D), the movement of specific nuclei could be tracked over the course of the recorded time (Figure 5E). We found that between bud and 5-somite stage (when the rostral neural plate is just completing closure), substantial movement can be observed in the ectoderm. Looking at the trajectories of the nuclei (Figure 5E), one sees that cells of the medial neural populations (basal plate) move anteriorly (green arrow), while lateral cells (alar plate) move either first towards the midline and then anterior (posterior alar forebrain, yellow arrow) or diagonally towards the anterior if they belong to the anterior alar forebrain (red arrow). These results therefore strongly suggest that the final location of the different diencephalic areas found in prim5 brains is taken during the beginning of somitogenesis, as the neural plate is “keeling.”

When we label cells of the midline (basal plate) in the neural plate, we observe often a more dorsal–positioned progeny of the cells then we first expected. A dramatic example of such observation is the presence of basal plate cells inside the ZLI (Figure 4F). In our uncaging experiments, we are not able to define the medio-lateral extent of the basal plate as there is no visible boundary between basal and alar neural plate. To find the border between basal and alar plate, we set out to mark solely basal-derived cells. It is known that, in zebrafish, cells of the basal plate originate close to the shield at 50% epiboly [25]. In contrast, cells of the alar forebrain are positioned in the animal pole region at the same stage. Therefore, we transplanted cells at 50% epiboly on top of the shield (Figure 5F and 5G) and selected at bud stage for embryos in which the transplanted cells were nicely spread along the midline (Figure 5H). By that approach, we labelled cells along the basal plate, similar to the uncaging experiment, but this time, because of the specific origin and movement of these basal cells during gastrulation, we were sure that just cells of the basal plate were marked. We let these embryos develop up to prim5 (Figure 5I) or prim22 (Figure 5J–5L) and compared the distribution of the transplanted cells to the expression of *shh*. Cells from the basal plate contribute to a bigger part of the rostral forebrain then we expected. In comparison to more caudal parts of the brain and spinal cord, basal plate cells can be found at much more dorsal positions in the rostral forebrain (up to the optic recess in its rostral-most portion), probably due to a high level of proliferation of these cells in the rostral central nervous system (CNS). Basal cells also contribute to the ventral part of the ZLI and thalamus (yellow arrows in Figure 5I and 5L). Just the tip of the ZLI (white arrows in Figure 5I and 5L) is always deprived of basal cells. In the hindbrain, very few cells can be found dorsal to the basal–alar border (arrowhead in Figure 5K), which are likely to reveal directed neuronal migration into the alar plate.

### The Ablation of the Anterior Diencephalon Anlage Results in Loss of the Prethalamus

Our fate-map experiments show that different forebrain territories, marked by the differential combination of gene expression, give rise to specific parts of the forebrain in later stages of development. This finding led us to address whether any of these territories is already functionally specified and irreplaceable.

**Figure 6:**
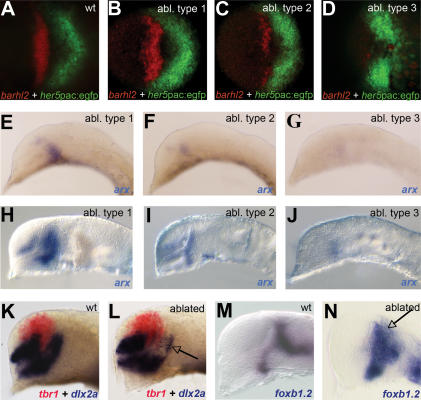
Ablation of Prethalamic Precursors (A–D) *barhl2* (red) and *her5*pac:egfp (green) are shown in (A) wild-type (wt) and (B–D) prethalamus-ablated (abl) embryos 2 h after surgery. (E–G) *arx* expression at the 12-somite stage and (H–J) prim5 stage in ablated embryos are shown, representing the different ablation types. (K and L) *tbr1* is shown in red, and *dlx2a* in blue; in comparison to the wt (K), the prethalamic expression of *dlx2a* is reduced in ablated embryos (arrow in [L]). (M and N) *foxb1.2* expression at prim 5 shows that mid-diencephalic domains in ablated embryos are not impaired although the expression appears unorganized in the thalamic domain (arrow in [N]).

We focussed our effort on the domain forming the future prethalamus (dark red domain in Figure 4). Cells of this area were ablated by mechanically removing them from the neural plate (see Materials and Methods). To control the specificity of our ablations, we performed RT-PCR on RNA extracted from prethalamic cells (30 embryos ablated). We tested for presence of transcripts of the anterior diencephalic marker *barhl2,* the telencephalic marker *foxg1* [26], and the midbrain marker *pax2a*. Although *barhl2* is readily amplified, neither of the two others is detected (unpublished data). We observed that half of the treated embryos show a reduction or loss of the marker *barhl2* (*n* = 8, Figure 6A–6D) 2 h after suction of the cells. Another set of ablated embryos, fixed at the 10–12-somite stage, shows a complete (3/11) or partial (4/11) loss of the prethalamus marker *arx* (Figure 6E–6G). Finally, 19 ablated embryos were left to develop to prim5 stage. About a third of these show a dramatic reduction of *arx* (*n* = 6/19, Figure 6H–6J). A loss or severe reduction could also be observed with other prethalamic markers such as *dlx2a* (Figure 6K and 6L). The ablation of the prethalamic precursors does not lead to a loss of thalamic identity (based on *foxb1.2* expression at prim5 stage; Figure 6M and 6N). These data demonstrate that ablation of the prethalamic anlage impairs the formation of the prethalamus, therefore strongly suggesting that this area is indeed specified and irreplaceable at that stage.

### The Precursors of the Anterior Diencephalon Keep Their Identity in an Ectopic Environment

**Figure 7:**
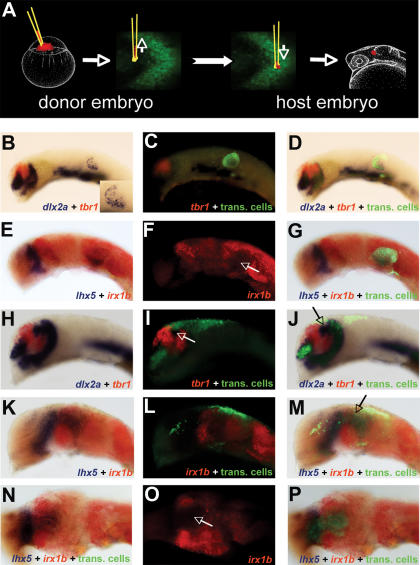
Transplantation of Prethalamic Precursors (A) Schematic overview of the transplantation experiment putting prethalamic precursors in ectopic regions of the neural plate. (B–D and H–J) shows *dlx2a* (blue), *tbr1* (red), and transplanted cells in green; (E–G and K–P) shows *lhx5* (blue), *irx1b* (red), and transplanted cells in green. (B–G) Transplanted cells located in the hindbrain show ectopic expression of the prethalamus markers *dlx2a* (B–D) or *lhx5* (E–G); no expression of the telencephalon marker *tbr1* (see inset in [B]) could be found in the clone. Cells do not exhibit expression of *irx1b* (arrow in [F]) in the transplanted cells. (H–P) After transplantation into the forebrain, ectopic cell–non-autonomous expression of *dlx2a* (arrow in [J]) could be seen (probably resulting in the split of the telencephalon, arrow in [I]) as well as of *lhx5* in the thalamus (arrow in [M]). Cells did not switch on the expression of thalamic *irx1b* (arrow in [O]) in the thalamus.

Having shown that prethalamic identity is already specified at bud stage, we assessed whether this commitment was sufficient to drive the development of these cells into differentiating progenitors. To address this, we tested whether these cells keep their identity and express later prethalamic markers in an ectopic location (Figure 7). Cells of the prethalamic anlage were therefore transplanted into ectopic regions of a host neural plate (Figure 7A). Around 75% of the transplants (*n* = 28) showed expression of the prethalamic markers *dlx2a* (Figure 7B–7D and 7H–7J), *lhx5* (Figure 7E–7G and 7K–7P), and *arx* (Figure S1). Because, from late somitogenesis onwards, these markers begin to be expressed in telencephalic progenitors, we analysed, in the same embryos, the expression of bona fide dorsal telencephalic markers *tbr1* [27] (Figure 7B–7D and 7H–7J) and *emx3* [28] (Figure S1), and show that the transplants acquired diencephalic, but not telencephalic characteristics. When cells are transplanted into the presumptive hindbrain region of the host neural plate (*n* = 8; Figure 7B–7G) we found expression of both *dlx2a* (Figure 7B–7D) and *lhx5* (Figure 7E–7G) inside the clone and no ectopic expression of the telencephalic markers *tbr1* or *emx3* (see inset of Figures 7B and S1). The absence of *irx1b* transcript inside the clones (arrow in Figure 7F) shows that these cells do not express the surrounding hindbrain characteristics. This finding indicates that the transplants develop as prethalamus, showing that these precursors, specified at bud stage, keep their identity independent of their location. In these cases, the transplants form round and compact structures, which are sometimes excluded from the hindbrain (Figure S1). In contrast to these results, donor cells located just rostral to the prethalamic precursors never express prethalamic markers when transplanted in ectopic neural plate areas (*n* = 7; unpublished data).

Contrasting with this observation, when cells are transplanted in more rostral regions (Figure 7H–7P), the clones are much more dispersed and donor cells are interspersed with the surrounding tissue (*n* = 7, Figure 7H–7J and unpublished data). In these cases, ectopic expression of our prethalamic markers is observed both in and outside of the clones (*n* = 5, Figure 7H–7M), sometimes splitting the expression of the telencephalic marker *tbr1* (arrow in Figure 7I). Ectopic expression of prethalamic markers could be observed anterior to the endogenous prethalamus, inside the telencephalon (Figure 7H–7J), or posterior, inside the thalamic area (Figure 7K–7P). As shown for the more caudal grafts, the rostral transplants do not express typical markers of the surrounding host tissue even when positioned inside the thalamus (arrow in Figure 7O).

Given the described role of the ZLI and its signalling component *shh* in maintenance of prethalamic identity [1,4], we tested whether *shh* signalling may play a role in the ectopic expression of prethalamic markers of our transplants. We treated embryos with the chemical cyclopamine, directly after transplantation. Even in embryos impaired for *shh* signalling, not only the endogenous *lhx5* expression was still detectable, but also the transplanted cells were still able to express this prethalamic marker (Figure S1G–S1I). The ability of the presumptive prethalamic cells to maintain their identity is therefore independent of the possible expression of *shh* by some of these precursors.

All together, these results unambiguously show that, in the neural plate, the presumptive prethalamic territory is strictly specified and is able to differentiate into an embryonic prethalamus in absence of any extrinsic contribution. Moreover, some prethalamic precursors are able to influence their environment, suggesting that at least a portion of these have the ability to impose prethalamic identity to presumptive forebrain cells.

## Discussion

Our current knowledge of the early diencephalic development is very limited. Studies done in fish, frog, chick, and mouse support the idea that, within the prospective forebrain, telencephalon and eyes are specified in regions of no or low Wnt activity, whereas posterior diencephalic fates are promoted by Wnt signalling [29–33]. However, the molecular, cellular, and temporal regulations leading to the acquisition of specific diencephalic identities (such as prethalamic, epithalamic, hypothalamic, thalamic, and pretectal) have yet to be elucidated.

We established an expression map for genes expressed in the anterior neural plate at bud stage. At such an early stage of development, we were able to find distinct expression territories within the diencephalon anlage. This result is the first experimental evidence showing that as soon as a diencephalic identity can be detected molecularly inside the neural plate, it already contains at least four distinct domains of gene expression. One cannot exclude the possibility that there may even be further discrete subdivisions. In this respect, it is worth noting that the boundary of expression of some of the markers is dynamic. This could create a further molecular difference at a slightly later stage in development, by establishing differential temporal exposure to various proteins. Our fate-map results show that the progeny of each labelled cell group develops in a continuous clone and does not show cell mixing with neighbouring regions. Clones could be allocated to distinct parts of the diencephalon, thus allowing us to predict which cells of the neural plate will develop into the prethalamus, thalamus, pretectum, and their basal counterparts. Forebrain fate maps done in mouse [7], chick [8,9], and frog [10] showed clonal distributions that suggest a similar lack of cell mixing. Knowing that a lot of morphological changes take place between bud and prim5 stage, such absence of cell mixing points to a considerable level of cohesion in cell movements. Indeed, our time-lapse imaging of the closing neural plate (Figure 5 and Video S1) revealed that the relative positions of the mapped prim5 forebrain territories are established during the formation of the keel, by a series of coordinated movements. In fact, most of the mapped neural plate territories adopted their relative positions by the 5-somite stage (completion of keeling).

Superimposing our expression and fate-map data, specific combinations of gene expression can be associated to specific fates. Interesting is the observation that some gene expression in the neural plate seems to be kept in the same cells up to prim5 stage, as shown for the example of *barhl2* (Figure 3C and 3D). In contrast, our work demonstrates that the neural plate *flh-*expressing cells (domain II), previously thought to include mostly the pineal gland precursors [18], form a territory contributing to the dorsal telencephalon, the roof of prosomere 3, and the epithalamus. Just very few cells of this early *flh* expression domain in the neural plate participate to form the pineal gland itself, although *flh* is clearly mostly expressed in the epiphysis at prim5 [18]. The fate map also reveals two origins for the ZLI precursors (defined by expression of *shh* [1]): a basal cell population forms the ventral part of the peak, and an alar cell population is contributing to the more dorsal ZLI. Our results support the model by which the ZLI forms at the interphase between an already specified presumptive prethalamus and thalamus. Although our data do not support the existence of a wedge-shaped precursor for the ZLI in zebrafish (see [1] for review), it does not disprove the existence of a ZLI compartment, although in fish, such a segment would have to be much narrower than in chick. The basal contribution to the ZLI is strongly supported by our transplants of basal plate cells. These transplants also highlight basal participation to extensive parts of the anterior forebrain, such as the pre-optic area and the ventral portion of the thalamus.

Our clonal analysis further shows that one is able to distinguish between prethalamic or thalamic precursors in the neural plate, pointing to the possible presence of a compartment boundary between these two populations at bud stage. This supports the presence of compartments suggested by the prosomeric model [3]. Our ablation and transplantation data indeed further suggest that the prethalamic territory acts as a compartment, with rostral and caudal boundaries, supporting the idea proposed in the latest prosomeric model [3], of a prethalamus acting as a boundary separating the secondary prosencephalon from the rest of the forebrain. It also indicates that such prosomeres may form during or just prior to neurulation.

Our ablation experiments show that the prethalamic precursors are irreplaceable and therefore are acquiring a unique cell identity between mid and late gastrulation. Detection of residual prethalamic identity at different stages following ablation uncovers some degree of recovery in a small proportion of the prim5 brains. This is likely due to a rescue of the area during the course of development in embryos in which prethalamic precursors were only partially ablated. More importantly, the fact that we still see a dramatic effect in a good proportion of the prim5 embryos indicates that the area cannot be rescued if all prethalamic precursors are removed. This finding indicates the presence of specific factors defining prethalamic identity. A handful of genes are to date known to be expressed in the anterior diencephalic neural plate. The combinatorial expression of these candidates may lead to the establishment of prethalamic identity. Future extensive expression profiling and loss of function experiments will elucidate the genetic components of such fate commitment. Very recent publications show that Fez and Fez-like are key proteins for prethalamic identity [34,35]. However, loss of function of these genes does not completely abolish induction of the prethalamic territory, indicating that other factors are required.

It has been shown that the expression of *shh* in the ZLI is important for maturation of the prethalamus [1,4,36]. One could argue that by ablation of the prethalamic anlage at bud stage, we partially ablate precursor cells of the ZLI, which are positioned close by and therefore may impair proper development of the prethalamus. However, we know that patterning genes such as *fezl* and *lhx5* are still expressed in the prethalamic anlage in the absence of *shh* expression in the ZLI (S. Scholpp and C. Houart, unpublished data, and Figure S1). Moreover, we do not observe a loss of *shh* expression in domain I–ablated embryos (unpublished data). The prethalamic identity is therefore lost in ablated embryos, despite the presence of *shh,* due to a loss of neural plate cells, which uniquely acquired a specific prethalamic competence.

Our transplantation experiments further confirm prethalamic fate commitment. Several hours following transplantation, prethalamic cells are still expressing markers specific to their fate. Strikingly, the transplanted cells not only keep the expression of the transcripts already present at the time of ablation, but are also able to pursue prethalamic differentiation. Indeed, these cells are able to express *dlx2a,* whose transcription starts to be induced in the wild-type diencephalon 6–7 h after bud stage. As mentioned earlier, *shh* plays a crucial role in the development of the prethalamus. Therefore, one could speculate that *shh* signalling may be involved in the establishment of prethalamic identity and activation of prethalamic markers in our transplantation experiments. We excluded this possibility because both endogenous *lhx5* transcripts in the developing prethalamus and ectopic expression in the prethalamic transplants are observed in embryos with impaired Hh signalling (Figure S1G–S1I). This finding demonstrates that the early steps in prethalamus development are not dependent upon Hh signalling and, therefore, that other factors are essential to establish the prethalamic fate within these cells of the neural plate.

When cells were transplanted away from their origin into the presumptive hindbrain, we observed a cell-autonomous induction of prethalamic markers and no induction of telencephalic markers. The result is rather more complex when transplants are placed in the presumptive forebrain. In these transplants, we observe cell–non-autonomous expression of the prethalamic transcripts *dlx2a* and *lhx5*. This surprising result suggests that some prethalamic precursors not only maintain their identity, but are also able to influence surrounding forebrain tissues. The prethalamic cells may secrete one or a combination of signalling factors, which can be interpreted by neighbouring forebrain tissue, but not by hindbrain cells. In our operated embryos, only a fraction of the transplanted domain is generally able to induce cell–non-autonomous changes, suggesting that a specific subset of the prethalamic precursors contains the signalling property. This signal is able to impose prethalamic identity to presumptive forebrain cells. In the wild-type situation, it is therefore likely that the action of this (these) presumptive signal(s) is restricted to a narrow region by formation of strict boundaries rostral and caudal to the prethalamic precursors. Here again, Hh is a potential candidate because Vieira and Martinez [37] observe some similar effects when transplanting quail Hh-expressing diencephalic basal plate into chick alar forebrain areas. However, if involved, it cannot be the only player, because our transplants ectopically induce *lhx5* expression, which is detected, even in wild type, much prior to ZLI formation and has been shown to be Hh independent (S. Scholpp, I. Foucher, N. Staudt, D. Peukert, A. Lumsden, and C. Houart, unpublished results; Figure S1).

It has been shown that signalling centres inside the rostral neural plate are important for the proper development of the forebrain. At the anterior neural border (ANB) WNT-inhibitors guarantee low *wnt* values in the rostral forebrain important for the development of the eye and telencephalon [30,38]. Expression of *fgf3* and *fgf8* is also described in the ANB [4,36], and FGF8 has been shown to be required for development of the rostral forebrain [39–41]. Finally, the midbrain–hindbrain boundary regulates the formation of the forebrain–midbrain border [42]. In this study, we show that precursors of the rostral diencephalon are strictly committed to their fate during gastrulation, may play a role as a key transition area, and are able to influence the surrounding forebrain territory. Future experiments addressing gene functions in these cells will identify the proteins responsible for their unique properties.

## Materials and Methods

### Whole-mount in situ hybridization and immunohistochemistry.

In situ hybridization and immunohistochemical stainings were performed according to standard protocols [4,20] The expression patterns of the following genes were visualized: *arx* [15], *barhl2* [17], *dlx2a* [43], *fezl* [16], *flh* [44], *foxb1.2* [45], *irx1* [46], *irx7* [19], *lhx5* [47], *shh* [4], *six3* [48], *pax6a* [20], *tbr1* [27], and *wnt8b* [49].

The GFP in embryos of the *her5*pac:egfp transgenic line was detected by using an anti-GFP antibody (Torrey Pines Biolabs, Houston, Texas, United States) and as a secondary antibody Alexa 488 (Molecular Probes, Invitrogen, Paisley, United Kingdom). The biotin in transplanted cells of Figure 7 was visualized with streptavidin 488 (Molecular Probes).

Photos were taken with a Nikon eclipse E800 microscope and figures made in Adobe Photoshop CS (Adobe Systems, Uxbridge, United Kingdom).

### Uncaging of fluorescein.

To label specific groups of cells in embryos of the *her5*pac:egfp transgenic line, 1% caged fluorescein (Molecular Probes) was injected in one-cell stage embryos. These embryos were incubated in the dark until bud stage. They were checked for their GFP expression and mounted in 5% methylcellulose. In cells of specific territories in the diencephalic neural plate, the caged fluorescein was converted via a laser beam (565 nm) to its fluorescent form. Subsequently, embryos were kept at 28 °C in the dark until they developed up to prim5 stage. Then embryos were fixed in 4% PFA for 3 h at room temperature or at 4 °C overnight. The uncaged form of the dye was visualized by using an anti-fluorescein antibody coupled with alkaline phosphatase (Roche, Basel, Switzerland), in some cases following in situ hybridization of marker genes.

### Time-lapse analysis.

A plasmid containing nuclear RFP (kindly provided by Megason and Fraser) was injected into *her5*pac:egfp transgenic embryos at one to four cell stage to get a mosaic distribution of the RFP-labelled nuclei. At bud stage, the embryos were embedded and oriented in 0.8% low–melting point agarose in Danieau medium. The agarose was overlaid with medium, and a time-lapse movie was made using a confocal microscope (Nikon eclipse C1; Nikon, Tokyo, Japan). Every 5 min, pictures were taken every 5 μm along the *z*-axis (170 μm in total) over a period of 10 h. Subsequently, the data were modified, and individual cells of the neural plate were tracked using Imaris 4.2 (Bitplane AG, Zurich, Switzerland) software.

### Cell ablation and transplantation.

Specific cells of the neural plate at bud stage were ablated by sucking these cells out with a manual syringe [6] (Sutter Instrument Company, Novato, California, United States). For transplantation, these cells were then placed in the neural plate of wild-type or *her5*pac:egfp host embryos. Partly embryos were treated with cyclopamine (100 μM; Toronto Research Chemicals, North York, Canada). The consequences of these manipulations were then revealed by detection of expression domains of various forebrain markers.

To follow the development of the basal plate, labelled cells were transplanted on top of the shield at 50% epiboly stage [26]. Once embryos reached bud stage, embryos showing a midline distribution of transplanted cells were further incubated up to 22 hours postfertilization (hpf) or 30 hpf.

## Supporting Information

Figure S1 Transplantation of Prethalamic Precursors in Ectopic Environment (A–C) At 22 hpf (dorsal view, rostral to the left), ectopic expression of *dlx2a* inside part of the clone cells expressing the prethalamic marker form an ectopic epithelial structure (arrow in [A]).
(D–F) At the 10-somite stage (lateral view, rostral to the left), ectopic expression of the prethalamic marker *lhx5* could be found in a subset of the transplanted cells, but no ectopic expression of the telencephalon marker *emx3* in that basally located clone was found.
(G–I) At 24 h, cyclopamine-treated from bud to 24 h, successful treatment with cyclopamine leads to the development of big and almost fused eyes ([G], asterisk); no hypothalamus is detectable. (H) Endogenous expression of *lhx5* (arrow) is still detectable in the prethalamus of cyclopamine-treated embryos. (I) Dorsal view, rostral to the left: ectopic expression of *lhx5* by the transplantation of anterior diencephalic cells after cyclopamine treatment (arrow) is shown.
(4.6 MB TIF)

Video S1 Movement of Nuclei in Early Stages of Neural Rod Formation Time-lapse analysis from early tailbud stage to 5-somite stage, dorsal view, anterior to the left. Mosaic labelling of nuclei with RFP (red) in *her*5pac:egfp (green) transgenic embryo.
(707 KB MOV)

## Abbreviations

GFP: green fluorescent protein
hpf: hours postfertilization
RFP: red fluorescent protein
ZLI: zona limitans intrathalamica
